# Dual-Manifold Contrastive Learning for Robust and Real-Time EEG Motor Decoding

**DOI:** 10.3390/s26061783

**Published:** 2026-03-12

**Authors:** Chengsi Hu, Qing Liu, Chenying Xu, Guanglin Li, Yongcheng Li

**Affiliations:** 1 School of Information Engineering, Guangdong University of Technology, Guangzhou 510006, China; 2 CAS Key Laboratory of Human-Machine Intelligence-Synergy Systems, Shenzhen Institutes of Advanced Technology (SIAT), Chinese Academy of Sciences (CAS), Shenzhen 518055, China

**Keywords:** brain–computer interface, EEG, manifold learning, contrastive learning, real-time processing, motor imagery

## Abstract

Brain–computer interfaces (BCIs) have great potential for consumer electronics, as they enable the decoding of brain activity to control external devices and assist human–computer interaction. However, current decoding methods for BCIs face several challenges, such as low accuracy, poor stability under electrode shift, and slow processing for real-time use. In this paper, we propose a hybrid decoding framework designed to address the challenges of current EEG decoding methods. Our method combines manifold learning with contrastive learning. The core of our method lies in a dual-manifold model that uses non-negative matrix factorization (NMF) and a contrastive manifold learning framework to extract clear and useful features from brain signals. To improve decoding stability, we introduce a joint training strategy that enhances feature learning. Furthermore, the system is optimized for real-time interaction, reducing the system latency to 100 ms. We collect EEG signals from 15 subjects performing motor execution tasks and 10 subjects performing motor imagery tasks to construct a motor EEG dataset. On this dataset, the proposed method achieves superior decoding performance, reaching F1-scores of 0.7382 for the motor imagery tasks and 0.8361 for the motor execution tasks. Furthermore, the method maintains robustness even with reduced electrode counts and altered spatial distributions, highlighting its potential as a decoding solution for reliable and portable BCI systems.

## 1. Introduction

Brain–computer interfaces (BCIs) have garnered increasing attention from researchers due to their promising potential. Decoding neural activity, they can be integrated into external devices such as exoskeletons or functional electrical stimulation systems. In particular, non-invasive BCIs based on electroencephalography (EEG) offer low-cost and safe deployment compared to their invasive counterparts. They offer new and suitable interaction pathways for all users [1,2,3].

However, electroencephalogram (EEG) signals are difficult to process. Owing to their weak signal intensity and strong non-stationarity, EEG-based BCIs are afflicted by randomness, nonlinearity, and heavy noise interference [4,5,6]. These issues are especially severe when decoding fine motor intentions. Low signal-to-noise ratios and artifacts such as eye movements or muscle activity significantly reduce classification accuracy.

In addition, most studies investigating EEG decoding methods focus on emotion recognition or short-term experiments, and few have explored cross-temporal continuous signal classification for motor execution and motor imagery [7]. In addition, existing methods often lack channel robustness. They rely too heavily on precise electrode placement, making them vulnerable to channel loss or displacement in real-world clinical settings [8]. Furthermore, current algorithms lack the temporal precision needed to capture the millisecond-scale dynamics of how motor intent emerges, which holds back the development of closed-loop BCI systems.

Accurately and rapidly decoding motor intent from EEG signals is important for BCI applications in real-time control scenarios. Over the years, researchers have proposed various methodologies, evolving from traditional handcrafted features to modern deep learning and manifold learning frameworks.

### 1.1. Traditional Decoding Methods

Early decoding methods relied heavily on linear algorithms and handcrafted features extracted from time, frequency, and time–frequency domains. For instance, González-Cely et al. [9] utilized the band power features from *α* (8–12 Hz) and *β* (13–30 Hz) rhythms based on the power spectral density (PSD). Similarly, Blankertz et al. [10] proposed the common spatial pattern (CSP) algorithm. This method improves class discriminability by maximizing the difference in spatial variance across motor-related channels. Although these approaches offer physiological interpretability and work well in standard BCI tasks, they struggle to capture the nonlinear and non-stationary neural dynamics often observed in complex motor tasks. Moreover, they generally require large time windows for stable estimation (often exceeding 1 s), making them unsuitable for real-time applications with millisecond-scale resolution needs.

### 1.2. Deep Learning-Based Approaches

With the growth of large EEG datasets and computational resources, deep learning has enabled the development of end-to-end decoding approaches. Lawhern et al. [11] developed EEGNet, a compact convolutional neural network (CNN) that captures spatio–spectral–temporal EEG features through depth-wise separable convolutions. Altan et al. [12] adapted novel lower–upper triangularization-based extreme learning machines (LuELMs) to the ConvNet architecture, improving its prediction ability, outperforming that of conventional fully connected neural networks with widely used spectral features. Garcia et al. [13] further demonstrated the feasibility of low-cost BCI systems through CNN–LSTM hybrid models. Recent studies have focused on enhancing decoding performance for robust BCI applications. For instance, Bunterngchit et al. [14] introduced GACL-Net, which is a hybrid deep learning framework that combines graph-attentive convolutional layers and LSTM networks. The network was evaluated on complex EEG data and achieved a classification accuracy of 99.52%, demonstrating its real-time processing capabilities and potential for practical applications. Additionally, Wang et al. [15] proposed IFNet, an interactive frequency convolutional neural network for enhancing motor imagery decoding by capturing frequency-specific features, showing improved performance on challenging datasets. Despite their advances, these approaches require EEG data with sufficiently large time windows for effective training. Moreover, most approaches assume stationary signal statistics and fixed channel configurations, which do not hold in actual BCI system design.

### 1.3. Manifold Learning and Modern Nonlinear Techniques

Manifold learning techniques, such as t-distributed stochastic neighbor embedding (t-SNE) and uniform manifold approximation and projection (UMAP), have gained popularity. These techniques assume that the main features of high-dimensional, non-stationary EEG signals actually reside on a lower-dimensional embedding, allowing algorithms to effectively unravel complex nonlinear geometric structures. Li et al. [16] proposed a parametric t-SNE approach leveraging time–frequency features from the discrete wavelet transform (DWT), improving motor imagery EEG classification through better visualization and nonlinear feature extraction. Du et al. [17] applied UMAP to motor intent recognition, showing its superiority over single-domain features. However, although manifold learning techniques effectively capture nonlinear dynamics, their high computational complexity and lack of generalizability limit their real-time application in practical scenarios, especially in environments with chaotic signal characteristics.

To address the abovementioned limitations, particularly the insufficient capability of existing methods to model neural dynamics in real time and their low robustness to spatiotemporal heterogeneity, we propose a hybrid decoding framework that integrates non-negative matrix factorization (NMF) with a contrastive embedding-based representation alignment dual-manifold collaborative modeling approach. The framework is validated on complex motor intent decoding tasks and combines physiologically grounded feature decomposition with adaptive representation learning.

- Dual-Manifold Collaborative Modeling: NMF extracts low-rank, neurophysiologically interpretable spatial representations from high-dimensional EEG signals, while contrastive manifold learning captures temporal embeddings that are robust to non-stationary cortical patterns. This combination ensures both interpretability and nonlinear separability in the learned feature space.
- Joint Optimization for Robustness to Sparse Channel Setups: A stage-wise optimization strategy is employed, where the encoder is first pre-trained with contrastive loss to learn discriminative representations, followed by joint optimization with cross-entropy loss to refine classification boundaries. This strategy significantly enhances robustness against sparse channel configurations. Experimental results demonstrate that it consistently outperforms both traditional algorithms and recent manifold learning methods, maintaining high stability even under limited electrode availability.
- Real-Time Efficiency: This framework achieves a decoding resolution of 100 ms. This duration is grounded in human–computer interaction (HCI) principles, as control latencies below 100–200 ms [18] are perceived as instantaneous by users, preventing system lag and motion sickness in BCI prosthesis control. Moreover, it does not sacrifice high-accuracy performance.

Experiments on data obtained from subjects performing motor imagery and motor execution tasks demonstrate reliable decoding performance, even under non-stationary conditions and strict latency constraints. Consequently, this work provides a practical foundation for real-time BCI-based interaction systems.

## 2. Materials and Methods

### 2.1. Data Acquisition and Preprocessing

#### 2.1.1. Data Acquisition

**Figure 1:**
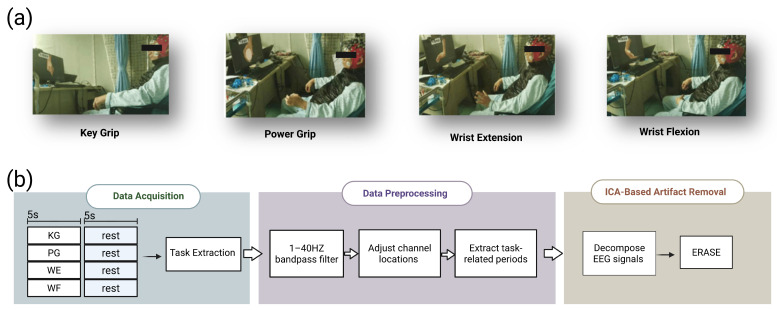
(**a**) Four paradigms: key grip (kg), power grip (pg), wrist extension (we), and wrist flexion (wf). The screen displays the relevant cues, and the subjects perform motor execution (ME) or motor imagery (MI) actions according to the cues. (**b**) Flowchart of the data acquisition and preprocessing steps, including data acquisition, preprocessing, and artifact removal.

The dataset comprises EEG recordings obtained from 25 healthy, right-handed subjects (13 males and 12 females, aged between 23 and 40 years). Specifically, 15 participants (9 males and 6 females) were asked to perform motor execution (ME) tasks, and 10 participants (4 males and 6 females) were asked to perform motor imagery (MI) tasks. All participants were required to possess normal or corrected vision and intact cognitive function, with no history of neuropsychiatric disorders and no recent use of central nervous system medications. All subjects completed two experimental sessions for each considered movement. This protocol allowed for a comprehensive comparison across different task modalities. Before the start of the experiment, the participants signed consent forms, and the research protocol was reviewed and approved by the Institutional Review Board at the Shenzhen Institute of Advanced Technology, Chinese Academy of Sciences, with IRB number SIAT-IRB-220715-H0601. We used a 64 channel Ag/AgCl EEG recording system (Compumedics Ltd., Abbotsford, VIC, Australia; Easycap GmbH, Wörthsee, Germany; Brain Products GmbH, Gilching, Germany), utilizing the international 10–10 electrode placement configuration (Figure 1a).

During signal acquisition, the ground electrode was placed at AFz, and the reference electrode was set to CPz. The sampling frequency was set to 1000 Hz, with electrode impedances maintained below 5 kΩ. All subjects were instructed to sit comfortably on a chair and to perform the motor tasks with their dominant hand following the video displayed on the computer screen (Figure 1a). We considered four essential motor tasks (Figure 1b). These tasks consisted of two grasping movements, key grip (KG) and power grip (PG), and two wrist movements, wrist extension (WE) and wrist flexion (WF). The video contained 10 images of active tasks (such as key grip) and 10 images of nonactive tasks (rest), totaling 20 motor tasks per video. As shown in Figure 1b (Data Acquisition), each active task in the video was displayed for 5 s, followed by a 5 s rest period to avoid mental fatigue. All subjects completed two experimental sessions for each considered movement. In addition, EMG signals were recorded from the bilateral frontalis, left and right temporalis to masseter, and bilateral trapezius muscles using the same acquisition system. The EMG signals were used as references for artifact removal from EEG signals, as detailed in the following section.

#### 2.1.2. Preprocessing

**Figure 2:**
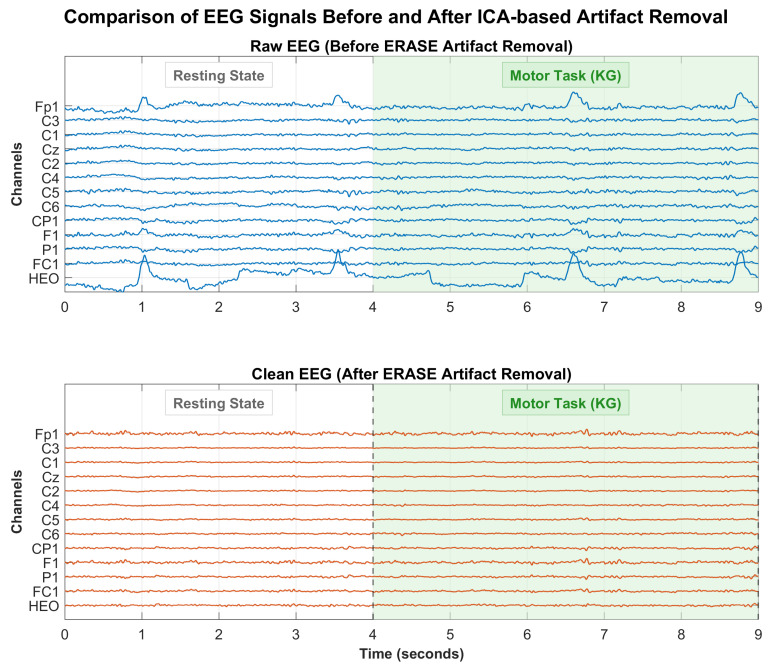
Comparison of EEG signals before and after artifact removal. The (**top**) panel displays the raw EEG data. The (**bottom**) panel shows the cleaned EEG signals. The green shaded region highlights the execution of the motor task.

EEG signal preprocessing was performed using the EEGLAB [19] toolbox and MATLAB R2023a. The preprocessing steps are demonstrated in Figure 1b. Specifically, the fourth-order Butterworth band pass filter with a cutoff frequency of 1 to 40 Hz was used to extract useful information from the EEG signals. Subsequently, the ERASE method [20] was employed to automatically remove artifacts (Figure 1b, ICA-Based Artifact Removal). This approach leverages the EMG signals collected from the face and head, the locations of which are described in Section 2.1.1, forcing the EMG artifacts to concentrate in a small number of independent components (ICs). The artifact-contaminated ICs were then identified and removed using an automated procedure, as demonstrated in Figure 2. After data cleaning, data splitting and temporal windowing were performed. Specifically, for each subject, a total of 20 trials were recorded for each class. To ensure a balanced class distribution, 16 trials per class were randomly selected for training, while the remaining 4 trials per class were used for testing. All processing was performed separately for each subject to conduct subject-specific evaluation. After data splitting, continuous EEG signals were split into 20 ms segments with a 50% overlap.

### 2.2. Method

#### 2.2.1. Non-Negative Matrix Factorization (NMF) for Feature Extraction

**Figure 3:**
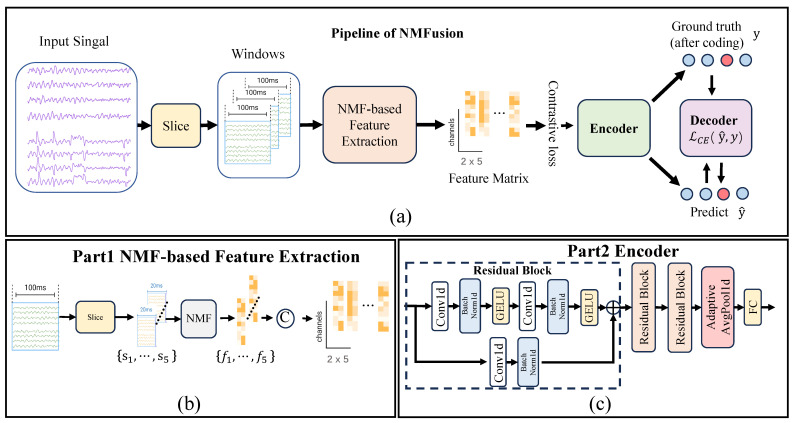
(**a**) Overview of the NMFusion architecture. (**b**) EEG windowing strategy. Each 100 ms window is split into five 20 ms segments. Features are extracted via NMF and then concatenated. (**c**) Encoder structure.

All processing was performed separately for each subject to conduct subject-specific evaluation. After data splitting, continuous EEG signals were split into 20 ms segments with a 50% overlap. We applied non-negative matrix factorization (NMF) [21] to extract meaningful spatial features from the EEG signals, with the complete processing pipeline illustrated in Figure 3a. The EEG data were first reshaped into a 3D matrix (channels × time points × trials), and absolute values were applied to ensure non-negativity. Each trial was then divided into 20 ms segments $X\in R_{+}^{C\times 20}$, where *C* is the number of channels. For each segment X, we solved the optimization problem,

(1) $$\min_{W,H\geq 0}{∥X-WH∥}_{F}^{2}$$

using multiplicative update rules,

(2) $$\begin{array}{cc} H_{aj} & \leftarrow H_{aj}\frac{{\left(W^{T}X\right)}_{aj}}{{\left(W^{T}WH\right)}_{aj}+\epsilon } \end{array}$$

(3) $$\begin{array}{cc} W_{ia} & \leftarrow W_{ia}\frac{{\left(XH^{T}\right)}_{ia}}{{\left(WHH^{T}\right)}_{ia}+\epsilon } \end{array}$$

with $\epsilon =10^{-9}$, where *i* indexes channels, *j* indexes time points, and *a* indexes components. Setting rank r=2 yielded spatial features $W\in R_{+}^{C\times 2}$ and temporal activations $H\in R_{+}^{2\times 20}$, with W retained as the feature representation. Five consecutive W matrices (representing 100 ms of neural activity) were concatenated column-wise,

(4) $$F={\mathrm{concat}}_{c=1:5}\left(W^{\left(c\right)}\right)\in R^{C\times 10}$$

creating a 10-dimensional feature matrix for each 100 ms block. Each F matrix was assigned a single label, and the aggregated features were finally standardized using subject-specific z-score normalization (zero mean, unit variance) computed across all motor tasks. The decoding temporal resolution of the model is equivalent to 100 ms (Figure 3a).

#### 2.2.2. Contrastive Manifold Learning

The contrastive embedding of biomedical recordings for analysis [22] is a contrastive learning framework designed to establish nonlinear mappings between high-dimensional neural dynamics and behavioral or experimental variables. Contrastive manifold learning operates in a supervised mode, using movement class labels (KG, PG, WE, and WF) to constrain the embedding space. To optimize temporal consistency, the model minimizes the contrastive loss function:

(5) $$L=E_{\begin{array}{c} x∼p(x),\\ y_{+}∼p\left(y|x\right) \end{array}}\left[-\psi \left(x,y_{+}\right)+\log\sum_{i=1}^{n}e^{\psi (x,y_{i})}\right],$$

where $y_{1},\ldots ,y_{n}∼q\left(y|x\right)$ denote negative samples, ψ(x,y) represents the similarity function, and $y_{+}$ is a positive sample from the conditional distribution p(y|x). This loss function ensures that the embeddings preserve the topological structure of the input data and yield robust representations of the neural dynamics feature.

#### 2.2.3. Improved Contrastive Learning Model

Based on the contrastive manifold learning framework, NMFusion’s architecture is a residual network (ResNet) [23] structure with a contrastive loss function to extract EEG features. It consists of a residual encoder, a contrastive loss function, and a joint training strategy. The encoder is a 1D convolutional structure with multiple residual blocks to facilitate deep network training. Each block contains two 1D convolutional layers, batch normalization (BatchNorm), and GELU activation. Skip connections are included to preserve feature information. The specific structure is as follows: The input layer is a 1D convolutional layer with a kernel size of 3, a stride of 1, and a padding of 1, producing 32 output channels, as shown in Figure 3b. Table 1 details the specific architectural parameters. This is followed by two residual blocks, each containing three layers with output channels of 32, 64, and 128, kernel sizes of 3, strides of 1 or 2, and a padding of 1. Finally, a global average pooling layer compresses the feature map into a fixed-length vector, which is then mapped to a 32-dimensional latent space through a fully connected layer. This design effectively captures the spatiotemporal features of EEG signals while avoiding gradient vanishing.

The goal of contrastive learning is to learn more discriminative feature representations by optimizing the similarity between positive pairs (from the same class) and negative pairs (from different classes) [22,24]. Positive pairs are randomly selected from samples of the same class, while negative pairs are randomly selected from samples of different classes. The similarity metric is based on the Euclidean distance, and the loss function includes an alignment loss and a uniformity loss, which enhance intra-class consistency and inter-class discrimination, respectively.

Based on the pre-training of contrastive learning, we propose a unique joint training strategy. This pipeline employs the cross-entropy loss function as a means of dynamically adjusting the decoding results of contrastive manifold training, thus optimizing both the encoder and classifier simultaneously [25,26]. The encoder first undergoes pre-training via the contrastive loss to learn time-domain feature representations. This process captures the similarities and differences between samples to construct a discriminative manifold feature space. Subsequently, the model enters a joint training phase. A fully connected layer maps latent features from the encoder to the class space. Optimization in this phase involves both contrastive and cross-entropy losses. This allows for the simultaneous refinement of feature representations and classification boundaries. By combining robust feature extraction with accurate classification, the strategy improves overall performance and stability.

The composite loss is defined as

(6) $$\begin{array}{cc} L_{\mathrm{contrastive}} & =E_{z,z_{+}}\left[-\psi \left(z,z_{+}\right)+\log\sum_{j=1}^{M}\exp\left(\psi (z,z_{j}^{-})\right)\right], \end{array}$$

(7) $$\begin{array}{cc} L_{\mathrm{CE}} & =E_{x,y}\left[-\sum_{c=1}^{N_{c}}I\left(y=c\right)\log p\left(c|x\right)\right], \end{array}$$

(8) $$\begin{array}{cc} L_{\mathrm{total}} & =L_{\mathrm{CE}}\left(f_{\mathrm{dec}}\left(f_{\mathrm{enc}}\left(x\right)\right),y\right) \end{array}$$

where the encoder $f_{\mathrm{enc}}\left(\cdot \right)$ is pre-trained to minimize $L_{\mathrm{contrastive}}$. The similarity function is as follows:

(9) $$\psi \left(z,z^{\prime }\right)=-{∥z-z^{\prime }∥}^{2}.$$

Let x be the input sample and *y* be the ground-truth label. The encoder $f_{\mathrm{enc}}\left(\cdot \right)$ maps x to a latent representation z. This embedding is compared to a positive sample $z_{+}$ and *M* negative samples $z_{j}^{-}$. The classifier $f_{\mathrm{dec}}\left(\cdot \right)$ processes the encoder output to produce softmax probabilities p(c|x) on $N_{c}$ classes. I(y=c) is the indicator function, which equals 1 if y=c and 0 otherwise. Finally, $\psi (z,z^{\prime })$ represents the negative squared Euclidean distance between two embeddings.

The framework allows for the flexible optimization of traditional hyperparameters alongside key structural parameters, such as the number of retained NMF components (i.e., the factorization rank *r*) and the sliding window size, to accommodate specific clinical scenarios and hardware constraints.

#### 2.2.4. Comparative Methods

To validate the proposed framework, we compared NMFusion with the existing lightweight model. Experimental consistency was strictly maintained by applying a 100 ms temporal window with a 50 ms overlap and allocating 80% of data, as standard, for training across all methods.

##### Traditional Machine Learning

- Invariant Time Domain Descriptor (invTDD): This combines six adaptive dynamic temporal features developed by Asogbon’s team [27] and incorporates dynamic force variation compensation through normalized EEG [28] or EMG activation mapping.

##### Deep Learning Benchmarks

- EEGNet [11]: This is a compact convolutional neural network designed for EEG-based BCI, and it introduces depth-wise and separable convolutions to construct an EEG-specific decoding model that encapsulates well-known feature extraction concepts. This architecture is noted for its ability to generalize across diverse BCI paradigms (such as SMR and MRCP) and maintain robustness even when limited training data are available.
- DeepConvNet [29]: This is a deep architecture network inspired by computer vision models. It consists of four convolutional blocks followed by max-pooling layers. It is designed to learn hierarchical features directly from raw EEG data and serves as a representative baseline for deep convolutional networks.
- Shallow Temporal CNN (ST-CNN): This is a lightweight temporal convolutional network optimized for short windows based on previous works [30,31]. It consists of 5 temporal convolutional layers (kernel sizes decreasing from 64 to 16) with fewer filters to reduce computational cost, followed by 30% dropout and ReLU activation.
- Lightweight LSTM (L-LSTM): This is a simplified bidirectional recurrent neural network designed for sequence modeling with minimal complexity [32,33]. It employs 128-unit bidirectional layers with a temporal attention mechanism to focus on key signal segments without the overhead of deep transformers.

Finally, we confirm the specific contribution of the proposed framework by comparing it to a manifold learning baseline.

- Contrastive Manifold Learning (CEBRA): We combined the contrastive manifold learning encoder (described in Section 2.2.2) with an support vector machine (SVM) classifier. A comparison with this method helps isolate the contribution of our proposed dual-manifold fusion strategy versus a standalone contrastive learning approach.

Implementation Details: The deep learning models (NMFusion, EEGNet, DeepConvNet, ST-CNN, and L-LSTM) were implemented in PyTorch (version 2.1.0) and trained on an NVIDIA RTX 4090 GPU (Santa Clara, CA, USA). All models used the Adam optimizer (learning rate = 1 × 10^−3^) and cross-entropy loss. The traditional InvTDD method employed an SVM classifier (RBF kernel, C=1.0, γ=0.1).

#### 2.2.5. Model Evaluation

The models’ performance was quantified using the following metrics: classification accuracy, which is the percentage of correct predictions, and macro-averaged F1-score, which balances precision and recall across all classes, as commonly used in multi-class classification tasks and confusion matrices (class-wise prediction distributions). In addition to accuracy and F1-score, Cohen’s Kappa coefficient (κ) was employed to evaluate the consistency between the predicted classification and the ground truth. Statistical analyses were performed to evaluate the significance of performance differences between the proposed NMFusion framework and the baseline methods. A one-way analysis of variance (ANOVA) was conducted on the classification metrics across all subjects. When the ANOVA indicated significant main effects, Tukey’s honestly significant difference (HSD) post hoc test was applied to perform pairwise comparisons between NMFusion and each competing algorithm. The significance level was set to α=0.05. In all relevant figures, statistical significance is denoted by asterisks: * p<0.05, ** p<0.01, and *** p<0.001.

#### 2.2.6. Ablation Study of NMFusion Components

An ablation study was conducted to evaluate the individual contributions of entropy-aware two-stage joint optimization and dual-stream NMF feature extraction. In this analysis, three configurations were compared: the full NMFusion model, which integrates both components; the variant that omits the cross-entropy refinement stage while retaining the NMF pathway; and the configuration that omits the NMF branch while retaining joint optimization. All models utilized identical preprocessing procedures and hyperparameters to ensure consistency.

#### 2.2.7. Channel Reduction Protocol

Channel robustness was assessed using a progressive reduction strategy based on the international 10–10 system [34]. We defined six subsets with varying electrode distributions, narrowing from full-head coverage to specific motor-relevant regions.

- Set 1 (60 channels): Included most electrodes of the 10–10 system, excluding the reference electrodes and EMG channels.
- Set 2 (36 channels): Removed the outlying electrodes, retaining the frontal (F), fronto-central (FC), central (C), centro-parietal (CP), and parietal (P) regions.
- Set 3 (29 channels): Removed the frontal electrodes, retaining the FC, C, CP, and P regions.
- Set 4 (22 channels): Retained only the C, CP, and P electrodes.
- Set 5 (13 channels): Focused on the central and CP regions, removing parietal sites.
- Set 6 (7 channels): Retained only the core central electrodes (C1 to C6), which are the regions most relevant for motor decoding.

This reduction process allowed us to assess the performance degradation and stability of each model under decreasing spatial information, simulating practical scenarios where the electrode count is constrained by clinical or hardware limitations.

## 3. Results

### 3.1. Result of Ablation Study on NMFusion Components

The results of the ablation experiment showed that (Table 2) the complete NMFusion model achieved an accuracy of 83.66% on the motor execution (ME) task and 73.89% on the motor imagery (MI) task. Removing entropy two-stage joint optimization reduced performance to 79.57% on the ME task and 72.01% on the MI task, while disabling the dual-stream NMF structure yielded an accuracy of 81.91% on the ME task and 73.62% on the MI task.

### 3.2. Decoding Accuracy

We compared EEG decoding performance on two tasks, that is, motor imagery and motor execution tasks performed by healthy subjects, using seven classification methods: NMFusion, CEBRA, L-LSTM, ST-CNN, InvTDD, EEGNet, and DeepConvNet. Performance was evaluated based on the accuracy and F1-score metrics.

**Figure 4:**
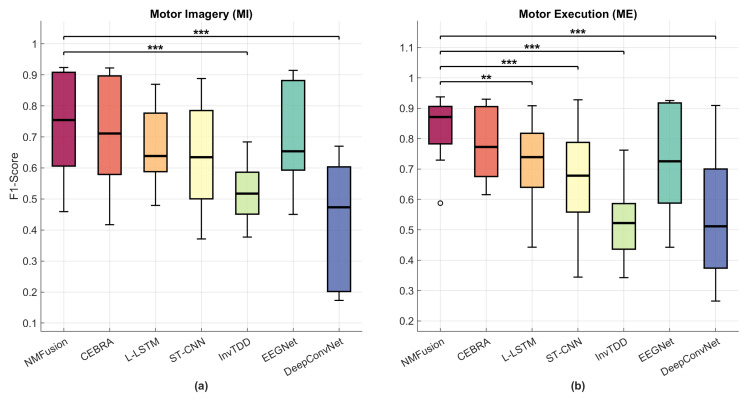
The F1-score distribution for different methods across two tasks: (**a**) motor imagery and (**b**) motor execution tasks. Each subplot compares the proposed NMFusion against the baselines, including CEBRA, L-LSTM, ST-CNN, InvTDD, EEGNet, and DeepConvNet. The boxes indicate the median and interquartile range (IQR). Statistical significance is marked as follows: ** p<0.01, *** p<0.001 (ANOVA with Tukey’s post hoc test). Color coding is consistent across all panels.

In the motor imagery decoding task (Figure 4a), NMFusion’s average accuracy was 73.89%, followed closely by EEGNet, which attained 73.08%, and CEBRA, which attained 72.01%. L-LSTM and ST-CNN achieved 68.22% and 65.84%, respectively, while DeepConvNet yielded 49.16%, and InvTDD obtained 52.70%.

**Figure 5:**
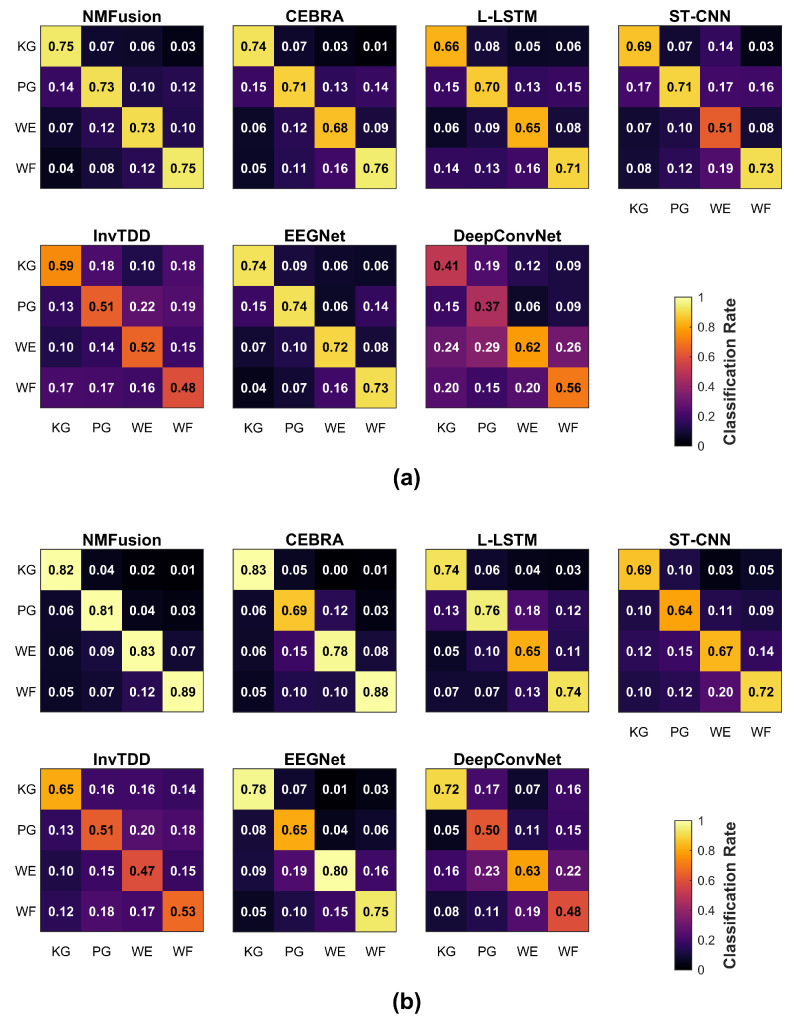
Average confusion matrices for healthy subjects performing (**a**) motor imagery (MI) and (**b**) motor execution (ME) tasks. The vertical axis represents the true class, and the horizontal axis represents the predicted class. The diagonal elements indicate the correct classification probability (recall) for each motor task, while the off-diagonal elements represent the misclassification rates. The color intensity corresponds to the prediction probability, allowing for a visual comparison of class-wise discriminability between NMFusion and baseline methods.

Across all subjects performing motor execution tasks (corresponding to Figure 4b), NMFusion achieved an average accuracy of 83.66%, followed by CEBRA at 79.57% and EEGNet at 74.42%. L-LSTM and ST-CNN obtained 72.26% and 67.69%, respectively. DeepConvNet and InvTDD yielded accuracies of 58.19% and 54.04%. The performance of these methods is further illustrated by the average confusion matrices, which visualize the classification results across different conditions and methods (Figure 5).

To provide a comprehensive evaluation of the decoding performance, Table 3 summarizes both the classification accuracy and Cohen’s Kappa coefficients (κ) across all subjects and tasks. As observed, NMFusion consistently demonstrated competitive or superior performance compared to the baseline methods in both metrics. Specifically, in the motor execution (ME) task, our method achieved a mean accuracy of 83.66% and a Kappa coefficient of 0.78.

### 3.3. Robustness to Channel Reduction

**Figure 6:**
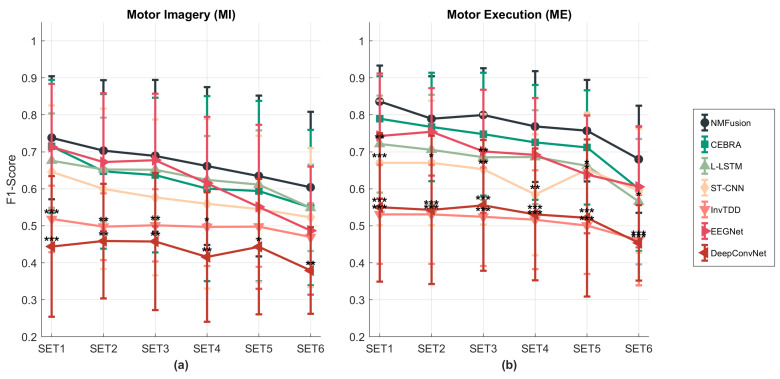
Performance comparison of different EEG electrode sets. (**a**) F1-score for healthy MI and (**b**) F1-score for healthy ME. Line plots with error bars show the mean F1-score ± standard deviation across different channel set configurations (SET1 to SET6). * p<0.05, ** p<0.01, *** p<0.001 (ANOVA with Tukey post hoc vs. proposed method). Marker shapes represent the methods (see legend).

As shown in Figure 6, the F1-score of each method changed as the number of EEG electrodes decreased. All approaches showed a downward trend, while EEGNet and ST-CNN exhibited notable fluctuations. Furthermore, InvTDD and DeepConvNet maintained consistently low F1-scores, leaving little room for further variation. The F1-scores of EEGNet and L-LSTM were competitive when the number of electrodes was 60 (SET1), yet their stability was poorer than that of NMFusion as the number of electrodes decreased. When the number of electrodes was reduced from 13 (SET5) to 7 (SET6), all methods experienced a drop. Nevertheless, NMFusion consistently outperformed all other approaches across channel configurations.

### 3.4. Computational Efficiency Analysis

As real-time processing is important for practical BCI applications, we compared the computational efficiency of the different approaches. As shown in Table 4, NMFusion achieved competitive training efficiency (64.8 s) compared to deeper models such as DeepConvNet (81.5 s). Furthermore, NMFusion’s per-sample prediction time was 0.044 ms, making it approximately 2.6 times faster than EEGNet (0.115 ms) and 3.2 times faster than DeepConvNet (0.140 ms).

## 4. Discussion

Our proposed framework has a manifold learning structure that integrates non-negative matrix factorization and contrastive learning. It encodes and decodes EEG signal features of exercise intention to address the challenges with regard to decoding accuracy and robustness in EEG-based BCIs. Our experimental data indicate that this method performs better than traditional lightweight approaches across all subject groups, particularly in motor execution tasks (with an F1-score of 83.61, as shown in Figure 4b). This result is similar to the findings of most related studies, such as those by Lee et al. [35].

In practical deployment scenarios, the number of target gestures directly affects the overall decoding performance of BCIs. Due to the low signal-to-noise ratio and limited resolution of EEG signals, fine kinematic movements often elicit highly overlapping cortical activation patterns [36]. Therefore, increasing the number of classifications will inevitably lead to a decline in classification accuracy and F1-scores. The four specific motor tasks selected in this study (key grip, power grip, wrist extension, and wrist flexion) achieve a balance between decoding reliability and functional utility. Clinically, these movements represent the most fundamental grasping actions required for activities of daily living, and they are also the primary target movements prioritized in stroke rehabilitation protocols [37]. Furthermore, from the perspective of neuroprosthetics and robotic control, these four intuitive intentions map directly to the basic degrees of freedom of an assistive robotic arm or hand exoskeleton, enabling control over spatial wrist orientation and end-effector grasping modes in practical use scenarios [38].

**Figure 7:**
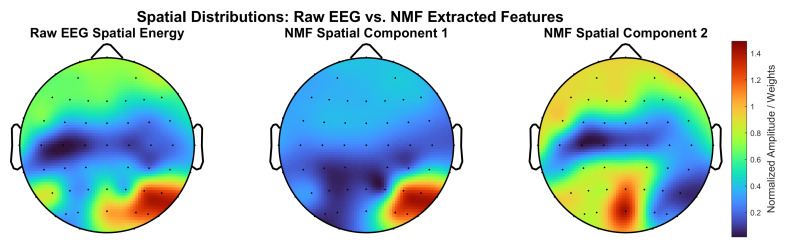
Topographical maps comparing the spatial energy distribution of raw EEG with the spatial weights extracted by the NMF module during the right-hand key grip task. Left: raw EEG spatial energy. Middle and right: NMF spatial components 1 and 2 (W).

Furthermore, the proposed NMFusion framework demonstrates neurophysiological interpretability. As visualized in Figure 7, the raw EEG spatial energy during right-hand execution exhibits a regional concentration over the motor cortex. Our NMF module acts as a spatial decomposer to mathematically extract these multi-channel signals into low-rank spatial basis vectors. Importantly, the extracted NMF spatial components explicitly encode localized neural activation patterns over the contralateral sensorimotor cortex. This confirms that the NMF module successfully extracts physiologically meaningful neural features of motor intentions rather than modeling peripheral noise or artifacts.

NMFusion’s dual-manifold structure is specifically designed for the limitation of standard deep learning models in simultaneously capturing both temporal dependencies and spatially invariant features. An ablation study (Table 2) confirmed that incorporating the preprocessing of NMF enables the model to extract spatial patterns relevant to neurophysiology, thus improving the accuracy of decoding. A critical challenge in real-time BCI development is balancing ultra-low decoding latency with the retention of essential physiological information. While classic markers such as event-related desynchronization (ERD) and synchronization (ERS) provide reliable signatures of motor intent, their extraction typically requires spectral power integration over extended temporal windows [39]. The proposed NMFusion framework provides an alternative perspective by exploiting the spatial dimensions of these neural phenomena. Neurophysiological studies have consistently demonstrated that brain activity is intrinsically parceled into discrete blocks of stable spatial patterns, known as EEG microstates, which typically last around 100 ms [40]. Furthermore, to effectively parameterize these states, our framework employs a fine-grained 20 ms segmentation strategy as a dense sampling mechanism, ensuring that the rapid, continuous spatiotemporal shifts of motor intentions are seamlessly captured.

Furthermore, in real-world scenarios, EEG signals often exhibit significant variability due to various reasons such as electrode displacement, impedance changes, or environmental noise [41,42]. These factors result in altered signal distributions and introduce artifacts, making traditional frameworks difficult to adapt effectively [43,44]. Our results (Figure 4) indicate that NMFusion effectively mitigates these issues by projecting neural signals to extract relevant features. As demonstrated by our channel reduction analysis, NMFusion exhibits exceptional adaptability and noise handling, maintaining robust classification even under sparse and shifting electrode configurations. NMFusion’s performance was assessed on datasets with varying electrode distributions, and it was found that the framework is able to extract specific neural dynamic features more stably and remains effective even when using only seven central electrodes. This mobility is highly advantageous for portable, wearable EEG applications outside controlled clinical settings. Additionally, a common challenge in prolonged BCI sessions is accumulated muscle fatigue, which typically causes amplitude shifts and frequency modulation. However, our dual-manifold contrastive learning framework inherently mitigates this physiological drift, ensuring robust classification even when fatigue alters the raw signal.

Moreover, its 100 ms temporal resolution satisfies the timing requirements for closed-loop neurofeedback, allowing for near-instantaneous feedback [45,46,47]. This duration not only complies with human–computer interaction (HCI) requirements [18] but also aligns with the brain’s natural information processing rhythm. In practical BCI use cases that require real-time performance, such as emergency grasping to prevent an object from falling or the rapid braking of a brain-controlled wheelchair, the low latency of our system can ensure safe and instantaneous physical interaction between the user and the device. Furthermore, in addition to physical neuroprosthetics, our method also provides a reference solution for application scenarios such as BCI-based virtual reality (VR) real-time interaction systems. Compared to traditional methods, which rely on rigid and high-density electrode configurations, NMFusion demonstrates significant possibility for portability and potential for consumer-grade BCI applications.

Compared to methods employed in recent motor imagery studies such as those by Lee et al. [35] and Cai et al. [48], our method demonstrates competitive or even superior performance. However, several limitations remain. The current NMFusion framework relies on subject-specific neural signatures to improve decoding performance [49], which may limit its immediate scalability. Furthermore, as highlighted in the computational analysis, our current latency metrics are derived from single-sample inference in an offline environment. We objectively acknowledge that a practical online BCI system introduces additional timing factors, including hardware data transmission, signal buffering, and dynamic online preprocessing. Specifically, incorporating an ICA-based artifact removal pipeline (such as ERASE) inevitably introduces computational overhead, which can bottleneck the overall speed of a truly real-time, end-to-end EEG system. For purely practical applications, one might consider omitting this rigorous artifact removal step entirely or substituting it with a lighter denoising algorithm. Nevertheless, the inclusion of ERASE in this current study is strictly necessary to guarantee the neurophysiological purity of the decoding process, proving that our framework achieves high accuracy by extracting genuine cortical motor features rather than peripheral artifacts. Future work will focus on exploring cross-subject commonalities; fully validating the end-to-end latency in closed-loop online experiments; and developing faster, hardware-friendly denoising schemes to achieve a fully optimal real-time BCI pipeline.

## 5. Conclusions

The proposed hybrid manifold learning framework (NMFusion) improves EEG-based motor intention decoding by effectively addressing signal non-stationarity and time latency. The experimental results demonstrate that NMFusion can extract neural features more stably than existing paradigms; additionally, it retains robust neural signal characteristics even under significant electrode reduction. Future research will prioritize practical system implementation, with a primary focus on achieving high-performance, real-time EEG decoding for everyday applications.

## Figures and Tables

**Table 1:** Detailed architecture and parameters of the NMFusion encoder.

Layer/Operation	Kernel/Stride	Output (C×T)
Input		
NMF Feature Matrix	-	$C_{in}\times 10$
Stem		
Conv1d	3×1 / 1	32×10
BatchNorm + GELU	-	32×10
Encoder Stage 1		
Residual Block $\times \;N_{1}$	3×1 / 1	32×10
Encoder Stage 2		
Residual Block $\times \;N_{2}$	3×1 / 2	64×5
(Shortcut: Conv 1×1)	1×1 / 2	
Encoder Stage 3		
Residual Block $\times \;N_{3}$	3×1 / 2	128×3
(Shortcut: Conv 1×1)	1×1 / 2	
Embedding		
Adaptive AvgPool	-	128×1
Flatten & FC1	-	128
FC2 (Latent)	-	32
Classifier		
Fully Connected (Out)	-	$N_{classes}$

Note: $C_{in}$: input channels; $N_{1,2,3}$: block counts; $N_{classes}$: number of tasks.

**Table 2:** Ablation study of NMFusion components (accuracy %).

Method	ME	MI
NMFusion (Full)	83.66	73.89
w/o Cross-Entropy Refinement	79.57	72.01
w/o NMF (Dual-stream)	81.91	73.62

**Table 3:** Comparison of classification performance on two tasks.

Method	Imagery (MI)		Execution (ME)	
	Acc (%)	κ	Acc (%)	κ
**NMFusion**	**73.89 ± 15.51**	**0.65**	**83.66 ± 9.06**	**0.78**
EEGNet	73.08 ± 14.23	0.64	74.42 ± 16.03	0.66
DeepConvNet	49.16 ± 15.87	0.32	58.19 ± 18.03	0.44
CEBRA	72.01 ± 16.51	0.63	79.57 ± 10.38	0.73
L-LSTM	68.22 ± 11.81	0.58	72.26 ± 12.45	0.63
ST-CNN	65.84 ± 16.40	0.54	67.69 ± 15.61	0.57
InvTDD	52.70 ± 8.54	0.37	54.04 ± 12.46	0.39

Note: MI: motor imagery; ME: motor execution. Best results are highlighted in bold. ACC: accuracy (%); κ: Cohen’s Kappa.

**Table 4:** Comparison of computational time of different methods.

Method	Feature Extraction and Training Time	Per-Sample Window Testing Time
NMFusion	64.8 s	0.044 ms
CEBRA	55.5 s	0.120 ms
ST-CNN	39.34 s	0.027 ms
L-LSTM	46.7 s	0.119 ms
invTDD	63.9 s	1.125 ms
EEGNet	46.2 s	0.115 ms
DeepConvNet	81.5 s	0.140 ms

Training on subject data was conducted on an NVIDIA RTX 4090 (Santa Clara, CA, USA) using PyTorch 2.1.0.

## Data Availability

Data are available on request due to restrictions (e.g., privacy, legal, or ethical reasons).
